# Mechanical and Surface Characteristics of Selective Laser Melting-Manufactured Dental Prostheses in Different Processing Stages

**DOI:** 10.3390/ma16186141

**Published:** 2023-09-09

**Authors:** Edgar Moraru, Alina-Maria Stoica, Octavian Donțu, Sorin Cănănău, Nicolae-Alexandru Stoica, Victor Constantin, Daniela-Doina Cioboată, Liliana-Laura Bădiță-Voicu

**Affiliations:** 1 Faculty of Mechanical Engineering and Mechatronics, National University of Science and Technology Politehnica Bucharest, 313 Splaiul Independentei, 060042 Bucharest, Romania; edgar.moraru@upb.ro (E.M.); grigore.dontu@upb.ro (O.D.); sorin.cananau@upb.ro (S.C.); nicolae.stoica@upb.ro (N.-A.S.); victor.constantin@upb.ro (V.C.); 2 The National Institute of Research and Development in Mechatronics and Measurement Technique, 6-8 Soseaua Pantelimon, 021631 Bucharest, Romania; cioboatadoina@yahoo.com (D.-D.C.); badita_l@yahoo.com (L.-L.B.-V.)

**Keywords:** dental prostheses, biomaterials, additive manufacturing, selective laser melting, indentation test, mechanical characteristics, microtopography, micro-/nano-roughness measurement, atomic force microscopy

## Abstract

Due to the expansion of the use of powder bed fusion metal additive technologies in the medical field, especially for the realization of dental prostheses, in this paper, the authors propose a comparative experimental study of the mechanical characteristics and the state of their microscale surfaces. The comparison was made from material considerations starting from two dental alloys commonly used to realize dental prostheses: Ni-Cr and Co-Cr, but also technologies for obtaining selective laser melting (SLM) and conventional casting. In addition, to compare the performances with the classical casting technology, for the dental prostheses obtained through SLM, the post-processing stage in which they are in a preliminary finishing and polished state was considered. Therefore, for the determination of important mechanical characteristics and the comparative study of dental prostheses, the indentation test was used, after which the hardness, penetration depths (maximum, permanent, and contact depth), contact stiffness, and contact surface were established, and for the determination of the microtopography of the surfaces, atomic force microscopy (AFM) was used, obtaining the local areal roughness parameters at the miniaturized scale—surface average roughness, root-mean-square roughness (RMS), and peak-to-peak values. Following the research carried out, several interesting conclusions were drawn, and the superiority of the SLM technology over the classic casting method for the production of dental prostheses in terms of some mechanical properties was highlighted. At the same time, the degree of finishing of dental prostheses made by SLM has a significant impact on the mechanical characteristics and especially the local roughness parameters on a miniaturized scale, and if we consider the same degree of finishing, no major differences are observed in the roughness parameters of the surfaces of the prostheses produced by different technologies.

## 1. Introduction

Metal additive technologies from the powder bed fusion (PBF) family open more and more opportunities for modern industries due to their ability to generate the most complicated metal parts with high precision and density in a relatively short time and enhance the final products in terms of geometrical features and in terms of some physical–mechanical properties. Selective laser depositions from the category of PBF additive technologies consist in applying a thin layer of powder with a fine granulation with a special leveling device, after which this powder bed is processed by means of a powerful laser beam or electron beam, melting spherical granules in the place of its projection [1,2,3]. The selective laser deposition group of technologies includes Selective Laser Sintering (SLS) [4], Direct Metal Laser Sintering (DMLS) [5], and selective laser melting (SLM) [6], which have a similar operating principle, the difference referring to the way the granules are bound. If in the first two mentioned technologies, powdered particles are processed without passing into the liquid phase (they heat up less, and sintering is the base) in the SLM process, more powerful lasers are used leading to the complete melting of the powder particles [1,2,3,7,8,9]. With the help of SLM technology, denser structures with better mechanical and surface characteristics are obtained due to the processing conditions and peculiarities, and the field of applicability is a wider one than in the case of other selective laser deposition technologies; but despite all this, the price of SLM equipment is generally higher than DMLS equipment, especially SLS equipment (which usually works with polymers or other non-metallic powders) [1,2,3,7,8,9]. All equipment control is performed with the help of a process computer in which the digital model of the future printing structure is loaded. This digital model is preprocessed in the software systems of the equipment in which sacrifice layers are created, scanning and selective processing trajectories are generated for all layers of the structure, important working parameters and specific processing parameters are set for processing a particular material, etc. [1,2]. Electron beam melting (EBM) is an additive processing method that belongs to the PBF category and has working principles similar to selective laser methods. In the case of EBM technology, electron beams are used instead of laser beams to selectively process powdered layers in a vacuum chamber, and only metallic conductive materials can be processed [10]. An alternative to PBF technologies is the metal additive technology, DED (Directed Energy Deposition) [11,12], in which the material in powder or wire form is delivered, melted (with the help of laser beam, electron beams, or arc plasma), and deposited at the same time on a substrate, somewhat similar to the classic additive technology, FDM (Fused Deposition Modelling) [13].

Some of the most frequently used raw materials for the family of PBF technologies are powders from alloys based on cobalt–chromium [14], nickel–chromium [15], various types of steel [16], aluminum [17], noble metals and some of their alloys [18], tungsten [19], titanium and its alloys [20], and powders from non-metallic materials (used more in SLS technology)—polymers [21], composites [22], or even ceramics [23].

The additive technology used to produce the dental prostheses in this paper is the SLM method, which has spectacular strengths and advantages [1,2,3,8,9,24,25,26,27] and is integrated into many important applications and offers new horizons for improving efficiency and capabilities in various fields [28,29,30], especially the medical field [31,32,33,34,35,36,37] and the dental field [1,2,38,39]. Even if the performance of dental prostheses made by SLM technology is worthy of appreciation, there are obviously some disadvantages; for example, the condition of initially obtained surfaces that are rough and require post-processing operations to obtain the right surface quality for the application in which they are provided [1,2,8,9,27]. The problem related to the poor quality of the initial surfaces obtained by selective laser melting technology is solved and compensated with the help of finishing [40] and mechanical [41] or electrochemical polishing methods [42]. These post-processing operations play a decisive role in the durability and future performance of dental prostheses [1,2]. First, there is a very close relationship between roughness and the mechanical properties of the prosthesis; rougher surfaces negatively affect wear resistance and fatigue characteristics, and it is common knowledge that cracks and other mechanical drawbacks occur on the surfaces of prostheses with higher roughness values, and even micro-level defects can contribute to the decrease in several mechanical features and affect the biofunctionality or may even cause the total failure of the prosthetic component [2,43,44]. Furthermore, there are also biological reasons to have a surface with the lowest possible roughness parameters because rougher surfaces facilitate the adhesion and retention of some inopportune and undesired microbial species that can affect the body with various local or even systemic medical problems [2,43,44]. Therefore, it is clear that the post-processing techniques of finishing and polishing the prosthetic components obtained through SLM technology have a fundamental and determining role in minimizing mechanical and biological threats and in increasing durability and mechanical characteristics and fulfilling the functional and anatomical role of the prosthesis [2].

With the advent and accelerated integration of additive manufacturing methods in the medical and dental fields, the scientific community has shown a constant interest in the research topic related to the properties of structures made by these technologies to evaluate, compare with classical technologies, and find viable solutions with the aim to improve the functionality and applicability of prosthetic components. For example, Øilo et al. [45] and Zhou et al. [46] comparatively researched the performances of the resulting mechanical characteristics for the structures made by SLM from a Co-Cr alloy with those made by classical casting and milling methods, and the results in both cases showed a significant influence of the production methods on the mechanical properties, and in the parts made by additive technologies, higher hardness values were obtained compared to the case of classic technologies. Han et al. [47] share the same opinion and conclude that better results can be obtained for dental prostheses using selective laser melting technology than traditional technologies. So, the superiority of SLM technology compared to the classic technologies for the realization of dental prostheses has been demonstrated, but this is also valued due to the corresponding post-processing finishing technologies that were used and contributed to the improvement of the roughness and the establishment of the respective final mechanical properties. Thus, the problem of the roughness of the parts made by SLM is of interest among researchers. Baciu et al. [48] analyzed the quality of the surfaces resulting from sand-blasting operations and observed how the mechanical and surface parameters improved after these operations, considering that 3D printed structures through SLM technology must be post-processed to be compliant for medical applications. In another work, Shu et al. [49] characterized the surface of dental implants made by selective laser melting both at the micro-level with the help of a 3D profilometer, and also at the nano-level with the help of atomic force microscopy.

Considering the lines discussed previously, the main objective of this paper is the evaluation of the mechanical properties and the zonal roughness parameters at the nano-scale level for the dental prostheses obtained by the PBF selective laser melting additive technology in the function of the material, execution, and post-processing technology. In order to assess some mechanical characteristics of dental prostheses, a standardized indentation test detailed in the next chapter was used, after which indentation hardness at specific applied test methodology, deformation level/penetration depths (maximum displacement/maximum indentation depth, zero displacement/permanent indentation depth, and contact depth), contact stiffness, and contact surface were determined. As a comparison term for the indentation test, dental prostheses made by SLM from two different materials, Co-Cr and Ni-Cr alloys and Co-Cr metal structure obtained by casting from a metal–ceramic dental prosthesis, were used. In turn, the dental prosthesis obtained by SLM was tested in two variants—preliminarily finished and polished condition. Regarding the establishment of parameters related to the quality of surfaces at the nano-scale, but also to establish the microtopography of the surfaces of dental prostheses executed by SLM, an AFM microscope was used following the values determined for *S_a_* (surface average roughness), *S_q_* (RMS roughness), and *S_y_* (peak-to-peak values—distance between the extremities of the irregularities). For this purpose, two dental prostheses made of a Co-Cr alloy obtained through SLM (finished and polished) and a dental prosthesis obtained through casting were used. Following the research carried out in this paper, conclusive and interesting results were obtained that demonstrate the strengths of the structures obtained through SLM in terms of mechanical properties and reflect the way in which post-processing operations influence the mechanical and surface characteristics of the dental prostheses made by selective laser melting. Also, mechanical characteristics values and parameters related to surface quality values were determined and compared from several perspectives: the material of the prosthesis (Co-Cr and Ni-Cr), the production technology (SLM and casting), and the stage of post-processing (finished and polished) for the appreciation of the mechanical characteristics, and the production technology (SLM and casting) and the stage of post-processing (finished and polished) to assess the zonal roughness parameters.

## 2. Materials and Methods

### 2.1. Materials and Technologies Used

**Figure 1:**
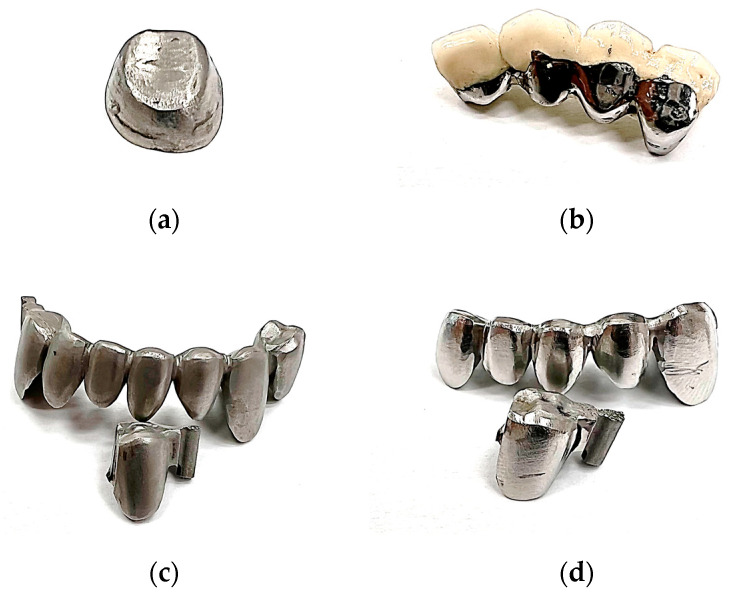
Dental prostheses used for indentation and surface test: (**a**)—finished SLM manufactured Ni-Cr dental crown; (**b**)—polished Co-Cr dental prosthesis obtained by casting; (**c**)—finished SLM manufactured Co-Cr dental prosthesis; (**d**)—polished SLM manufactured Co-Cr dental prosthesis.

As discussed in the previous chapter, comparative experimental research was performed from three perspectives: depending on the material, the realization technology, and the post-processing technology. Therefore, a total of 4 denture specimens were used for the mechanical indentation tests (Figure 1) as follows:

- Dental crown made by SLM from a Ni-Cr alloy in a preliminary finished state (Figure 1a).
- The mechanical substructure made of a Co-Cr alloy within a metal–ceramic prosthesis, the post-processing state is in polishing conditions and it is practically ready for functional use (Figure 1b).
- Complex prosthesis consisting of several dental structures made by SLM from a Co-Cr alloy in a preliminary finished state. For the mechanical tests, a unitary specimen was used that was sectioned from the entire prosthesis in order not to affect the compliance of the samples and fix them better on the experimental stand platform (Figure 1c).
- Complex prosthesis consisting of several dental structures made by SLM of a Co-Cr alloy in a polished state. For the same reasons as in the case of the finished Co-Cr prosthesis, a unitary specimen of the dental structure was used, which was sectioned from the entire prosthesis (Figure 1d).

The dental prostheses presented in Figure 1 were executed at an anatomically accurate scale based on the teeth of a real-life patient.

In the case of the tests regarding the microtopography and the quality of the surfaces of the prosthetic components, the same three Co-Cr prostheses were used (the two manufactured by SLM and one by casting); the Ni-Cr dental crown did not intervene in the study because it was considered more evaluation of the prostheses made by SLM regarding the quality of the surfaces depending on the post-processing stage and the comparison with the prostheses made by classical methods. As comparison criteria, we have prostheses made of two different materials, Co-Cr and Ni-Cr, with the same degree of finishing (only for mechanical tests), prostheses made by different technologies, SLM and casting with the same degree of finishing from almost the same material (tests mechanical and surface), prostheses to which different degrees of finishing/post-processing were applied, and finished SLM prosthesis and polished SLM prosthesis (mechanical and surface tests).

The dental prostheses used in this study were made by SLM technology and are based on fine-grained powders of alloys based on Co-Cr (tungsten, molybdenum, and silicon are also contained in the alloy) and alloys based on Ni-Cr (molybdenum, silicon, niobium, and aluminum are also contained in the alloy). After completion, considering the quality of the resulting initial surfaces that left much to be desired, which is a phenomenon specific to powder bed fusion type additive technologies, post-processing operations were gradually applied to them, such as preliminary finishing and mechanical polishing with fine abrasive particles. More details regarding the process of making and post-processing SLM dental prostheses, as well as other particularities, can be found in some previous studies [1,2,50]. Regarding the prosthesis made by classical methods, casting technology was used, and appropriate post-processing operations were used in order to be able to apply the ceramic layer and be ready for biofunctional conditions.

The dental prostheses made with SLM additive technology from materials used in this study were analyzed from the point of view of the elemental composition with the help of scanning electron microscopy (SEM) and energy-dispersive X-ray spectroscopy (EDS) by means of the Thermo Fisher Scientific (Waltham, MA, USA) Phenom ProX analysis system because the structures made do not always fully correspond to the composition specified in the technical data sheet of the raw material [1,2].

**Figure 2:**
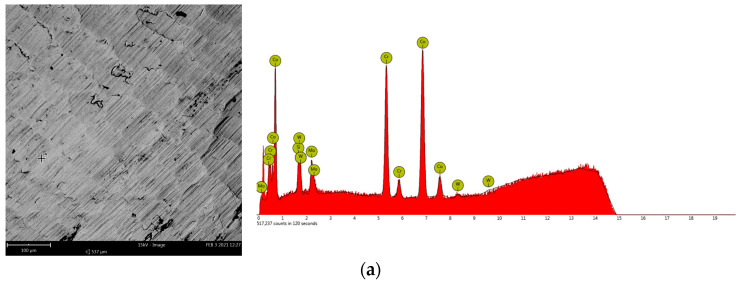

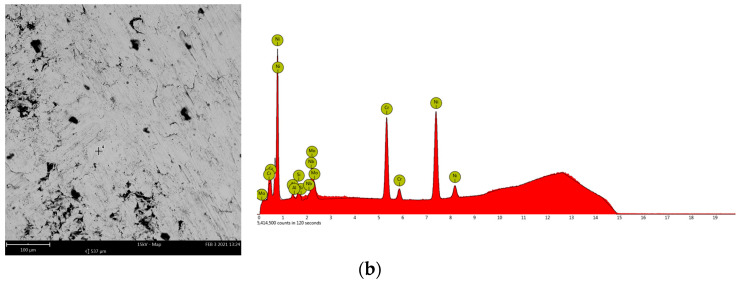
SEM images and EDS results of dental prostheses samples realized via selective laser melting: (**a**)—Co-Cr sample; (**b**)—Ni-Cr sample.

Figure 2 represents SEM images (magnification 500×) of one of the investigated points together with the corresponding EDS results for Co-Cr and Ni-Cr dental prostheses realized via SLM [2]. Table 1 and Table 2 present the obtained results regarding the average atomic and mass concentrations with one standard deviation (SD) established in six different points on the surface of the cobalt–chromium and five different points on the surface of the nickel–chromium SLM dental prostheses using elemental analysis by EDS spectroscopy [2,51,52].

### 2.2. Indentation Test of Dental Prostheses

**Figure 3:**
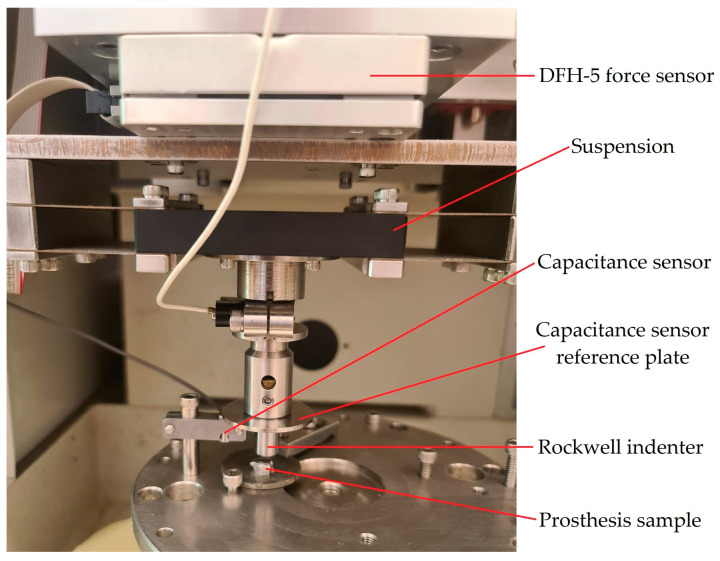
UMT II Multi-Specimen Test System setup.

Indentation testing, a well-known procedure, was used to determine the mechanical characteristics of the dental prosthesis samples. The tests were performed on a Bruker (Billerica, MA, USA, former CETR (Campbell, CA, USA)) UMT II Multi-Specimen Test System. The UMT II machine was equipped with a DFH-5 2-dimensional force sensor that has a range of 0.5 to 50 N and a resolution of 2.5 mN. In addition, a suspension for the DFH model force sensor was used to maintain the loading force as stable as possible. To measure the displacement, a capacitance sensor with a range of 254 μm and a resolution of 0.01 μm was used. The testing rig setup is presented in Figure 3.

**Figure 4:**
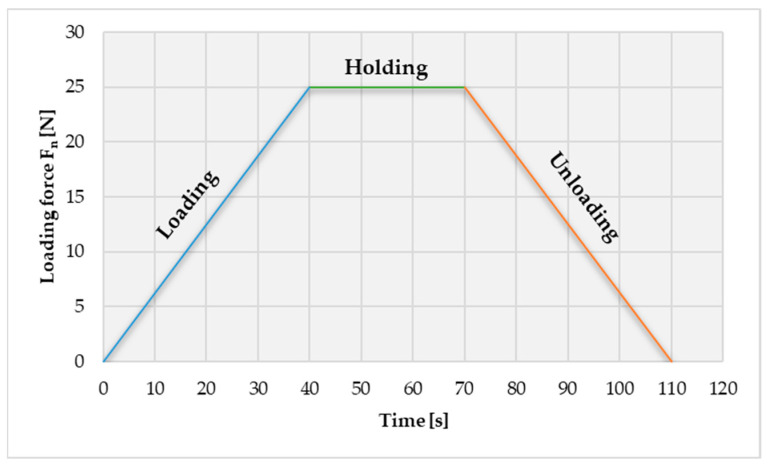
Applied indentation test methodology for dental prostheses.

The Indentation tests were performed based on the guidelines of the ISO 14577-1:2015 standard [53]. For these tests, a Rockwell diamond spherical tipped conical indenter that has a 120° angle and a 200 μm radius was used. The selected testing procedure was force-controlled and consisted of three steps, as seen in Figure 4. During the first step, which has 40 s and is called the loading step, the indenter penetrates the material while the applied normal loading force increases constantly for 0 to 25 N. The second step is called the holding stepIse the force is kept constant during a 30 s period. In the third and last step, called the unloading step, the force decreases constantly from 25 to 0 N over a 40 s period. The test force, $F_{n}$, the corresponding indentation depth, h, and testing time were recorded during the whole test procedure. All tests were performed at a temperature of 25 °C. To minimize errors, three tests were performed for each sample.

Based on the normal force and the indentation depth, the post-processing software of the UMT II Test System (Data Viewer 2.16) can automatically determine the indentation hardness ($H_{IT}$), the maximum displacement, the zero displacement, the contact depth, the contact area, and the contact stiffness ($S=dF_{n}/dh$).

The indentation hardness, which is a measure of the resistance to permanent penetration or damage, is calculated as [53]:

(1) $$H_{IT\;\;}=\frac{F_{max}}{A_{p}\left(h_{c}\right)}\;,$$

where $F_{max}$ is the maximum indentation force, $A_{p}\left(h_{c}\right)$ is the projected (cross-sectional) contact area between the indenter and the test sample, and $h_{c}$ is the depth of the contact of the indenter with the test sample at $F_{max}$.

### 2.3. Surface Characteristics Measurement of Dental Prostheses

Given the influence of denture surface roughness on mechanical characteristics and durability over time, but also biological considerations, issues discussed in Section 1, such as the dental prostheses used in this study, were investigated from the point of view of microtopography, and the main roughness parameters were determined.

For this purpose, we used dental prostheses made by SLM technology from Co-Cr in the finished and polished states to observe how roughness parameters vary depending on the degree of post-processing, but also the Co-Cr metal substructure of the metal–ceramic prosthesis realized through classical casting technology by comparing and analyzing whether the method of obtaining has an impact on the condition of surfaces.

**Figure 5:**
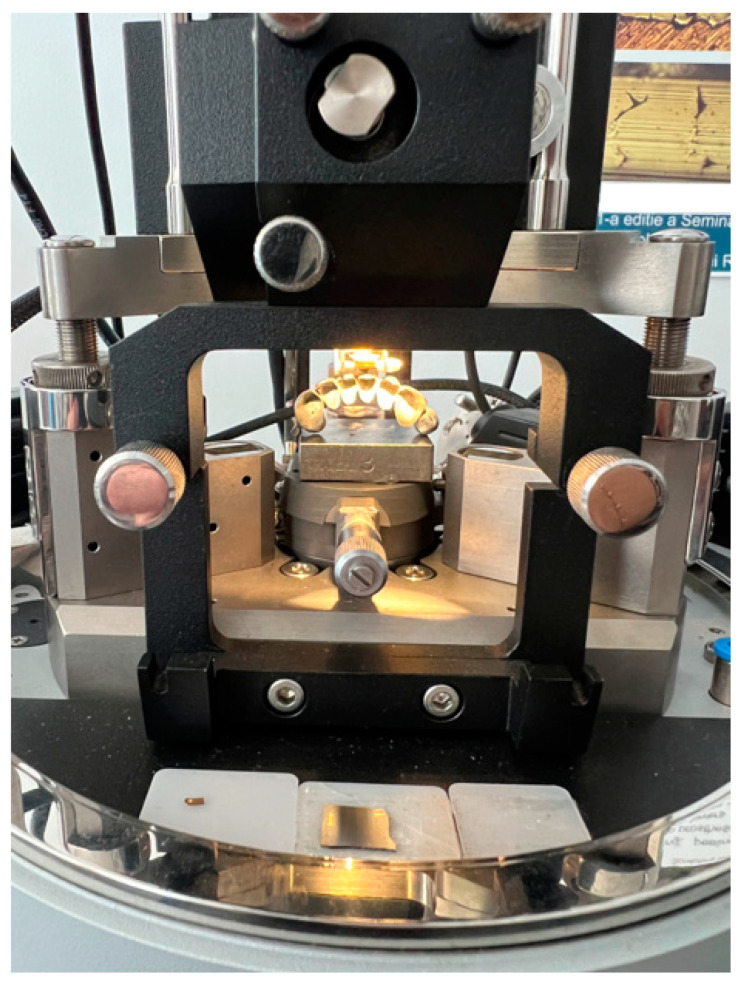
Dental prosthesis on the work platform of an AFM microscope.

The microtopographic analysis and determination of the main roughness parameters of the dental prostheses took place with the help of an NT-MDT Spectrum Instruments (Limerick, Ireland) NTEGRA Probe NanoLaboratory AFM microscope; the prosthesis on the equipment platform used is presented in Figure 5. This type of microscopy is based on determining the force between a small tip and the area of interest whose roughness parameters are to be established using a cantilever with a sharp tip at the end, and the force acting on the tip after interaction with the area of interest causes the cantilever to bend. By determining the deformation of the cantilever, it is possible to establish the force that occurs at the interaction between the peak and the evaluated area, and by writing down and processing these small deflections of the cantilever, surface topographies can be made by means of the atomic force microscope [2,54,55].

The aim of the research is to determine the zonal–local condition of surfaces through microtopography and the nano-roughness of dentures and assess how technology and the post-processing stage influence these aspects at a miniaturized scale. These determinations were based on choosing and scanning 100 × 100 μm areas (from an initial global 800 × 1000 μm area) from different regions on the surface of the 3 prostheses in the study, and then the parameters of interest were evaluated and processed by the AFM equipment software (Nova software). The parameters of interest refer to the areal parameters of roughness: average roughness (*S_a_*), the equivalent arithmetical mean height (*R_a_* parameter of a line) for the surface that expresses the average roughness of the absolute ordinate (vertical) Z axis in a given area (*x, y*); the root-mean-square parameter of roughness, the *RMS* (*S_q_*) of ordinate (vertical) values in a given area; and the distance between the extremities of irregularities, namely the distance between the highest and the lowest point in a given area, the peak-to-peak value (*S_y_*). Equations (2)–(4) show the relationships with which the previously discussed roughness parameters can be calculated, where *A* is a given area [56,57].

(2) $$S_{a}=\frac{1}{A}\underset{A}{\iint }\left|Z\left(x,y\right)\right|dxdy$$

(3) $$S_{q}=\sqrt{\frac{1}{A}\underset{A}{\iint }Z^{2}\left(x,y\right)dxdy}$$

(4) $$S_{y}=max\left(Z\left(x,y\right)\right)+\left|min\left(Z\left(x,y\right)\right)\right|$$

It should be mentioned that the selection of relatively smooth areas at this scale and the avoidance of areas with considerable surface microdefects from the global area initially scanned for all the surfaces of the investigated prosthetic components were taken into account, the final goal was not necessarily to find out the roughness parameters at a global level but at the zonal–local level, and practically to determine the roughness parameters close to the minimum ones in the respective analyzed area and thus see what impact post-processing technologies have at a miniaturized scale by taking into account the problems that may arise due to surface microdefects or biological reasons [2,43,44]. To obtain more conclusive results, three determinations were performed for each sample in different areas of the prostheses.

## 3. Results

### 3.1. Results of the Mechanical Characteristics of Dental Prostheses Obtained after an Indentation Test

Based on the indentation tests, we were able to determine the following mechanical properties of the investigated dental prostheses: indentation hardness, $H_{IT}$ 25/40/30/40 (where 25 is the test force, in Newtons, 40 is the application time of test force, in seconds, 30 is the holding time of the test force at maximum test force, in seconds, and 40 is the time taken to remove the test force, in seconds), deformation level after indentation, and three parameters of penetration depth—maximum displacement (maximum indentation depth at maximum applied force), zero displacement (permanent indentation depth, which remained in the structure after the removal of the load), and contact depth (depth of the contact of the diamond indenter with the examined structure at the maximum applied load), and also contact stiffness and contact area. Four samples were tested: a dental prosthesis made by SLM from a Ni-Cr alloy with a finished surface, a dental prosthesis made by SLM from a Co-Cr alloy in polished conditions and one with a finished surface, and a prosthesis made from a Co-Cr metal structure obtained by casting from a metal–ceramic dental prosthesis. The tests were performed three times for each sample, and the results are presented in Table 3, where the average value was calculated for each parameter. Regarding the results obtained for the indentation hardness determined under the conditions described above, an average value of 0.841 GPa was obtained for the dental prosthesis made of Ni-Cr in the finished state made by selective laser melting and a comparable value of 1.636 GPa and 1.683 GPa for the average hardness values were obtained for the polished Co-Cr cast prosthesis and the finished SLM Co-Cr prosthesis, respectively. In the case of the dental prosthesis made by SLM in a polished state, a higher average hardness value was obtained—2.252 GPa. For the resistance to indentation deformability, the maximum deformation reached (maximum displacement), the remaining deformation after releasing the load (zero displacement), and the depth of contact of the indenter with the specimen (contact depth) were determined. Regarding the maximum displacement achieved in the structure, the lowest value results from the polished prosthetic component made by selective laser melting—the average value for the three tests was 14.872 μm, which was the most resistant to deformability among the investigated structures. For the other prosthetic components, we obtained average values for maximum displacement as follows: 35.41 μm for the finished Ni-Cr SLM prosthesis, 20.699 μm for the polished Co-Cr cast prosthesis, and 20.154 μm for the finished SLM Co-Cr prosthesis.

In terms of zero displacement, or residual deformation, things are about the same but with much closer differences for the values obtained for the dental prostheses used in the study: 17.908 μm for the finished Ni-Cr SLM prosthesis, 7.116 μm for the polished Co-Cr cast prosthesis, 6.746 μm for the finished SLM Co-Cr prosthesis, and 6.372 μm for the polished SLM Co-Cr prosthesis. Regarding the contact depth results, we obtained the following results: 25.153 μm for the finished Ni-Cr SLM prosthesis, 12.624 μm for the polished Co-Cr cast prosthesis, 12.163 μm for the finished SLM Co-Cr prosthesis, and 9.198 μm for the polished SLM Co-Cr prosthesis.

The lowest average contact stiffness is obtained for the finished Ni-Cr SLM prosthesis—1.756 N/μm, and the highest value for the polished SLM Co-Cr prosthesis—3.315 N/μm. For the polished Co-Cr cast prosthesis and the finished SLM Co-Cr prosthesis, almost equal values are obtained again—2.331 N/μm and 2.338 N/μm, respectively. And finally, for the contact area, the following average values resulted: 26,619.788 μm^2^ for the finished Ni-Cr SLM prosthesis, 15,357.766 μm^2^ for the polished Co-Cr cast prosthesis, 14,818.635 μm^2^ for the finished SLM Co-Cr prosthesis, and 11,287.993 μm^2^ for the polished SLM Co-Cr prosthesis.

**Figure 6:**
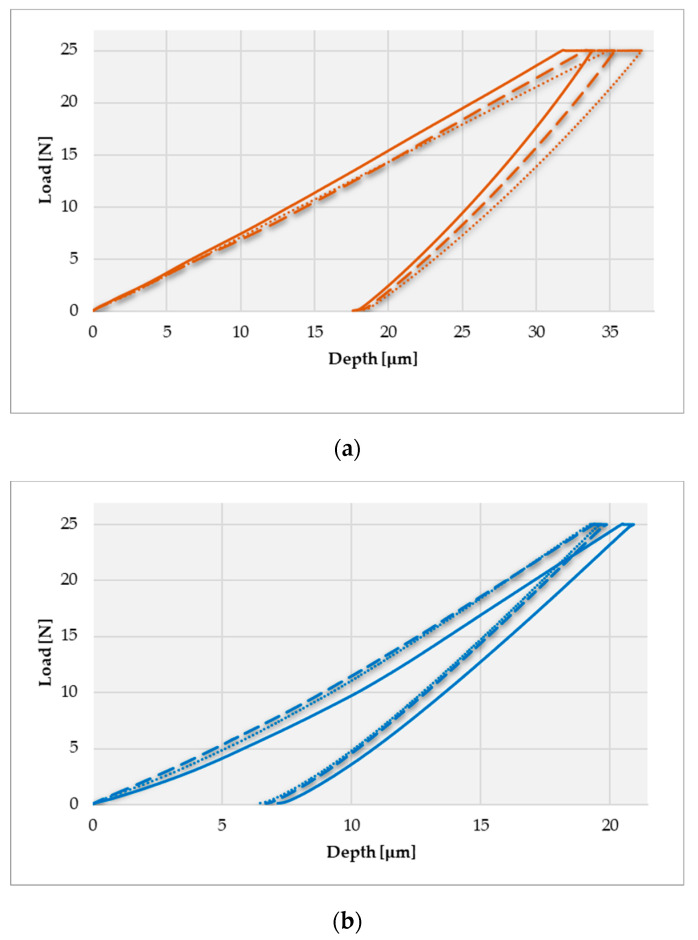
Indentation depth displacement behavior of SLM-manufactured dental prostheses in the finished state—Ni-Cr (**a**) and Co-Cr (**b**) in three different zones (differentiated according to the style of the curved line).

**Figure 7:**
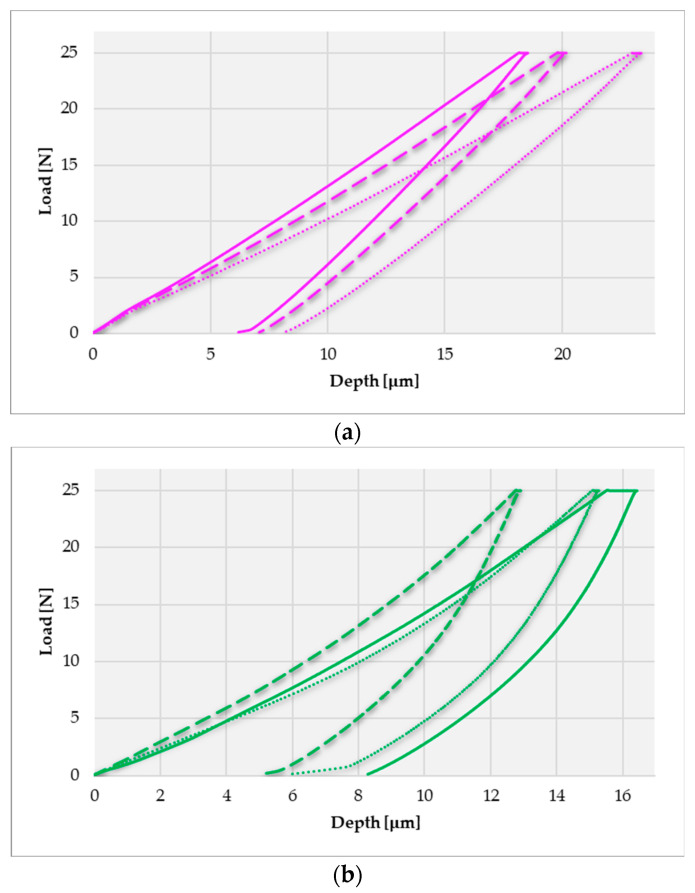
Indentation depth displacement behavior of dental prostheses in the polished state—Co-Cr obtained by casting (**a**) and Co-Cr (**b**) obtained by SLM in three different zones (differentiated according to the style of the curved line).

Also, based on the research carried out, we determined the indentation behavior of the deformation depending on the applied load according to the methodology described previously and presented in Figure 4. Figure 6 shows the indentation depth displacement behavior of the SLM-manufactured dental prostheses in a finished state in all three investigated zones, and Figure 7 presents the indentation depth displacement behavior of the dental prostheses in the polished state in all three investigated zones (the three tests are differentiated according to the style of the curved line—a continuous line for the first test, a broken line for the second test, and a dotted line for the third test).

As can be seen in the graphs, there are three distinct areas: when the deformation increases with the force up to 25 N (loading step from research methodology), a smaller area where the deformation increases even though the force remains constant at 25 N (holding step from research methodology), and at the end of this step the maximum displacement value is obtained, and an area where the deformation decreases as the force decreases (unloading step from methodology), finally reaching the residual deformation (zero displacement or permanent indentation depth value). Both in the graphs discussed in this chapter and the following comparative graphs in the Discussions chapter, the same chromatic highlight rule will be kept: brown color for the finished Ni-Cr SLM prosthesis characteristics, purple color for the polished Co-Cr cast prosthesis characteristics, blue color for the finished Co-Cr SLM prosthesis characteristics, and green color for the polished Co-Cr SLM prosthesis characteristics. More details regarding the interpretation of the results and the observations made following the indentation tests and the determination of some mechanical characteristics for dental prosthetic components can be found in the Discussions chapter.

### 3.2. Microtopography and Zonal Nano-Roughness Results of Dental Prostheses Obtained by AFM

As stated in Section 2.3, the main purpose was to determine the topography and areal roughness parameters at the zonal–local level. Therefore, in the initial phase, an area of 1000 × 800 μm was scanned on the surfaces of the dental prostheses, from which later, through the AFM microscope Nova software, different surfaces of 100 × 100 μm were selected on which the surface evaluations were made. These dimensions of 100 × 100 μm were a criterion applied to all denture surfaces in this study. The equipment program can give us information about the state of the surfaces in 2D format, where the differentiation of higher or lower regions is performed on a color scale, as well as in 3D format, in which the topography of the surfaces can be generated with details about the relief and the distribution of irregularities on the respective.

**Figure 8:**
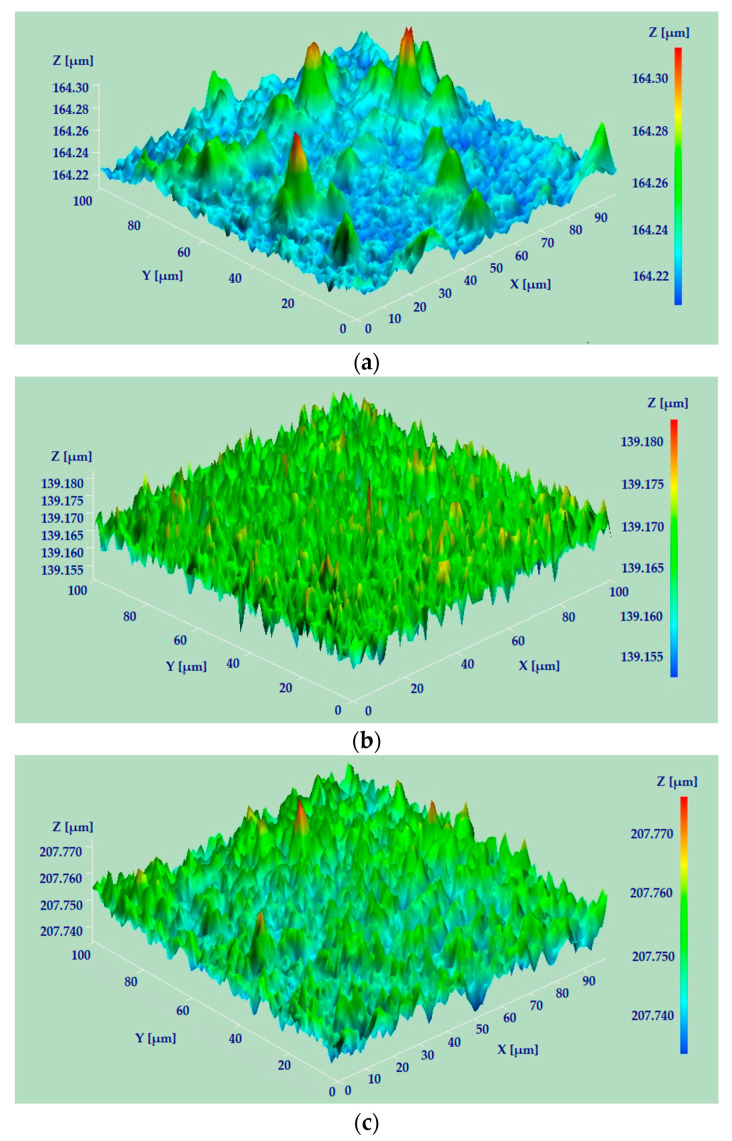
Obtained microtopography of dental prostheses on an AFM microscope: (**a**)—finished SLM manufactured Co-Cr dental prosthesis; (**b**)—polished SLM manufactured Co-Cr dental prosthesis; (**c**)—polished Co-Cr dental prosthesis obtained by casting.

Figure 8 shows the microtopographies obtained for some areas of 100 × 100 μm (X and Y axes) on the surfaces of the investigated dentures; the surface irregularities were identified on the Z axis. The high peaks are much more pronounced for the SLM dental prostheses in the finished state (Figure 8a) than the polished dental prosthetic components. There are no major differences between the microtopographic images obtained for the polished SLM prosthesis (Figure 8b) and the cast prosthesis in a polished state (Figure 8c), except that perhaps at the prosthetic structure made by selective laser melting, a smaller difference between the highest and lowest regions on the surface and a more uniform and dense distribution of irregularities on the surface can be observed.

It should be mentioned that the reference point for each investigated area is not zero, it depends on the placement of the prosthesis on the measuring platform of the equipment, and this aspect can be seen in the values on the Z axis from the microtopographic images. This does not affect the values of the established roughness parameters; basically, the difference between the highest and the lowest value on the Z axis is actually the peak-to-peak value (*S_y_*).

Also, following the measurement on the AFM microscope, information can be generated regarding the average profile on the X and Y axes, the histogram of the measurement, and the values of the determined roughness parameters. Table 4 shows the results obtained for the main parameters from zonal nano-roughness determinations on the AFM microscope. In addition to the results illustrated in the table, other parameters can also be determined on the AFM microscope: the maximum and minimum point in the investigated area (the difference between them being the *S_y_* parameter), ten-point height value (*S_z_*), surface skewness (*S_sk_*), coefficient of kurtosis (*S_ka_*), and other parameters related to the condition of the surfaces [2,54,55].

All the results obtained and illustrated in Table 4 were rounded to two decimal places. Therefore, after calculating the average of the values obtained for the three areas considered in this study, the following results regarding the zonal–local roughness can be highlighted. In the case of the finished SLM prosthesis, there was an average roughness of 7.75 nm, an average RMS value of 11.40 nm, and an average peak-to-peak value of 123.76 nm; in the case of the polished SLM prosthesis, there was an average roughness of 3.42 nm, an average RMS value of 4.25 nm, and an average peak-to-peak value of 32.53 nm; in the case of the polished cast prosthesis, there was an average roughness of 3.53 nm, an average RMS value of 4.54 nm, and an average peak-to-peak value of 42.10 nm. The results obtained regarding the microtopography and nano-roughness of the surfaces of dental prostheses provide interesting conclusions regarding the impact of post-processing operations on the quality of the resulting surfaces, and discussions on this topic are developed in the next chapter.

## 4. Discussions

### 4.1. Mechanical Characteristics

**Figure 9:**
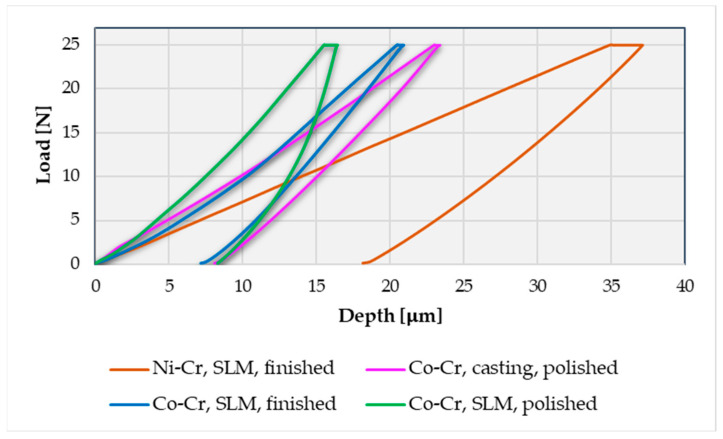
Comparison of indentation load-depth (displacement) curves for the dental prostheses in the most affected zone.

A comparative graphical analysis of the load-depth curves for the indentations with the highest displacement (the most affected zone by the indentation) for the four prostheses samples can be seen in Figure 9. These curves indicate a visco-elasto-plastic behavior for all four samples, with an emphasis on the polished SLM Co-Cr sample. Furthermore, it can be clearly seen that the Ni-Cr sample has a much higher deformation than the Co-Cr samples, both in terms of maximum displacement (the penetration depth at the end of the holding stage) and zero displacement (the residual plastic deformation at the end of the unloading stage). Also, the Ni-Cr prosthesis shows the greatest increase in indentation creep deformation (indentation deformation during the holding step). For the Co-Cr samples, it can be also highlighted that plastic deformation is not significantly influenced by post-processing operations, but the elastic deformation (difference between zero and maximum displacement) is. The polished SLM sample has a lower elastic deformation than the finished ones and the casted one, indicating that it is the hardest to deform.

**Figure 10:**
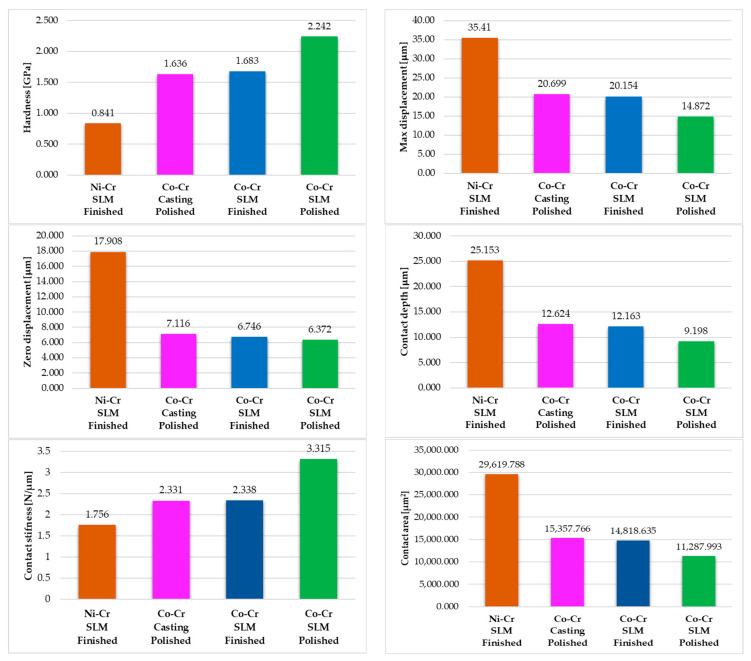
Comparison of the average values of the mechanical characteristics obtained after indentation tests.

To better analyze the results, a graphical comparison of the average values of the mechanical characteristics obtained from the indentation tests was performed and can be seen in the diagrams presented in Figure 10. As expected, the Co-Cr samples, due to the presence of tungsten in their composition, have better mechanical properties and are deformed less than the Ni-Cr one.

By comparing the average results for the finished and polished SLM prostheses, we can see that the post-processing operation applied to the surface has a significant impact on the indentation behavior of the material. Polishing the surface of the prosthesis leads to a 33.2% increase in hardness and a 41.8% increase in the contact stiffness compared to the finished surface. Regarding the indentation deformations, we can observe that the polishing of the SLM dental prosthesis compared to the preliminarily finishing process has the following effects: a 26.2% decrease in the maximum displacement, a 5.5% decrease in the zero displacement, a 24.4% decrease in the contact depth, and a 23.8% decrease in the contact area.

The method by which the samples are made influences their mechanical properties. This can be seen by comparing the average results of the polished SLM Co-Cr sample and the casted Co-Cr sample. The SLM prosthesis has a 37% higher hardness and a 42.2% higher contact stiffness than that obtained through casting. Furthermore, the polished SLM prosthesis deforms significantly less than the traditionally manufactured one: a 28.1% lower maximum displacement and a 10.5% lower zero displacement, and a 27.1% lower contact depth and a 26.5% contact area than the polished casted one. In terms of mechanical properties and deformation, the polished casted Co-Cr sample has results similar to the finished SLM sample with comparable values for all investigated parameters.

If we consider the same manufacturing technology and the same post-processing step (finishing) by analyzing the two different materials, Ni-Cr and Co-Cr alloys, it can be observed that the Co-Cr alloy has a double indentation hardness and a 33.1% higher contact stiffness compared to the Ni-Cr alloy. Also, in terms of deformation, the Co-Cr sample has a 43.1% lower maximum displacement, a 62.3% lower zero displacement, a 51.65% lower contact depth, and a 50% lower contact area.

After the interpretation of the results of the indentation tests, the superiority of the SLM sample over the casted sample has been demonstrated in terms of mechanical characteristics due to specific particularities of the manufacturing process (the effect of laser layer processing during the selective laser melting technology can also be considered a thermal treatment that enhances some mechanical properties). Moreover, it was shown that the post-processing operations have a major impact on the behavior of the samples during the indentation test and improve their mechanical characteristics.

### 4.2. Microtopography and Zonal Nano-Roughness

As can be seen in the microtopographic images (Figure 8), the post-processing operations have a significant influence even at this miniaturized scale. Compared to the dental prostheses after the last post-processing operation (polishing) before the application of the ceramic layer, the finished dental prosthesis shows, in the area of 100 μm × 100 μm, major differences between the height of the irregularities and has well-pronounced high peaks. In the case of both polished dental prostheses, one made by additive technology and one by classical casting technology, the result is a surface with a much smoother and denser distribution without significant differences between the extremities of the irregularities. Practically, the polished prostheses have an almost identical distribution of irregularities, perhaps with a slight superiority of the SLM-polished prostheses, observing a slightly smoother and more homogeneous distribution and a slightly smaller difference between the peaks compared to the surface of the casted prosthesis.

**Figure 11:**
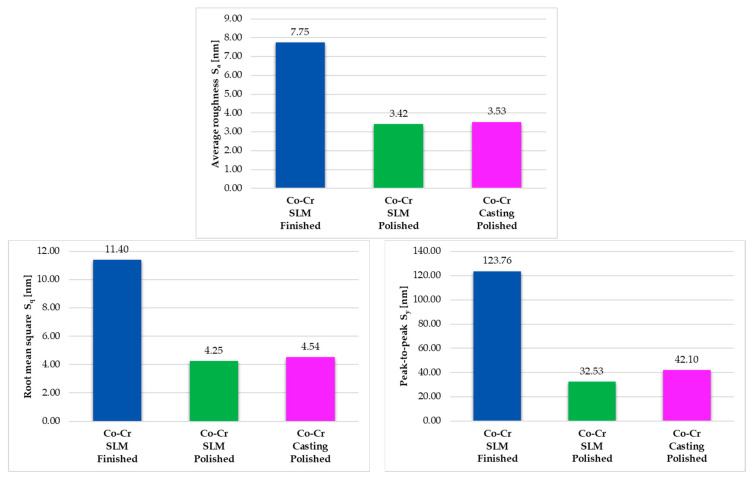
Comparison of surface parameters’ average values obtained after zonal nano-roughness determinations on an AFM microscope.

Figure 11 compares the results of the surface parameters’ average values obtained after zonal nano-roughness determinations on an AFM microscope. Significant differences are observed between the values obtained for the preliminarily finished prosthesis surface and the polished prosthesis surfaces. Regarding the average roughness, it turns out that polishing improves this parameter at this scale more than twice (average values of *S_a_* were 7.75 nm for finished SLM prosthesis, 3.42 nm for polished SLM prosthesis, and 3.53 nm for polished cast prosthesis), and in the case of an RMS, over 2.5 times (average values of RMS were 11.40 nm for finished SLM prosthesis, 4.25 nm for polished SLM prosthesis, and 4.54 nm for polished cast prosthesis). If we are discussing peak-to-peak values, differences are even more accentuated (average values of *S_y_* were 123.76 nm for finished SLM prosthesis, 32.53 nm for polished SLM prosthesis, and 42.10 nm for polished cast prosthesis).

It seems that the method of obtaining does not have a major impact on the state of the resulting surfaces, as long as the same mechanical polishing process is applied. The major differences occur between the surfaces in different stages of post-processing. Relatively comparable values were obtained in terms of average roughness and RMS for the polished prostheses made by SLM and casting. However, there are relatively small differences in the *S_y_* parameter, which refers to the distance between the extremities of the irregularities, where the average value of this parameter was almost 10 nm lower in the case of the dental prosthesis surface obtained by SLM, but it should be noted that in the case of the prosthesis made by casting, the closest results were obtained for the three studied areas (the difference between the maximum and minimum value for roughness parameters) in the three prostheses under study.

Taking into account that the surface roughness of dental prostheses, including at the miniaturized scale, influences their performance from biological considerations or mechanical properties, this study is useful for evaluating the efficiency of the post-processing operations at the micro- or nano-level and for selecting the optimal process or the eventual correction of the one used [2,43,44,48,49,50].

## 5. Conclusions

Based on the research carried out in this paper, the following can be concluded:

- Considering the same technology (SLM) and the same degree of finishing, compared to the Ni-Cr prosthesis, the Co-Cr one had a twice higher average indentation hardness and a contact stiffness over 33% higher. The Co-Cr prosthesis was also harder to deform than the Ni-Cr one.
- Considering the same degree of finishing and almost the same material (Co-Cr alloy), the prosthesis realized via SLM has a 37% higher indentation hardness and more than 42% higher contact stiffness than the prosthesis obtained by casting. Also, the indentation deformations are much smaller in the case of the prosthesis made by SLM than in the case of the prosthesis obtained by casting.
- The best mechanical properties and the greatest resistance to deformation after indentation tests were obtained in the case of the polished SLM dental prosthesis. The preliminarily finished SLM dental prosthesis has mechanical properties comparable to the polished one made by casting.
- Both in the case of the mechanical properties and especially in the case of the condition of the surfaces, the major impact that the post-processing operations of dental prostheses have on these characteristics was shown. After polishing the dental prosthesis made by SLM, a significantly higher average indentation hardness was obtained compared to the results obtained for the preliminarily finished SLM dental prosthesis (2.242 GPa compared to 1.683 GPa) with a greater contact stiffness (3.315 N/μm compared to 2.338 N/μm). Also, the polishing of the prosthesis led to an increase in resistance to deformation; for example, an average maximum displacement of 14.872 μm was obtained for the polished sample compared to an average maximum displacement of 20.154 μm for the preliminarily finished sample. The effects of polishing were even more evident for the average values determined for the microscale roughness parameters: in the case of the polished SLM dental prosthesis, the average roughness improved more than twice, the RMS parameter more than 2.5 times, and the distance between the extremities of the irregularities decreased almost 4 times compared to preliminarily finished SLM dental prosthesis.
- The roughness parameters are not significantly influenced by the method of obtaining the dental prostheses after applying the same degree of post-processing. Obviously, the differences appear when moving to a higher degree of finishing.
- Surface roughness plays a decisive role in durability and mechanical properties, but also in avoiding possible problems of biological nature; therefore, the use of appropriate post-processing operations for dental prostheses is very important.

As possible research perspectives, we can mention the study of other materials, technologies, or other post-processing methods for dental prostheses and also use other analysis and characterization methods and equipment.

## Figures and Tables

**Table 1:** Average atomic and mass concentrations with a standard deviation of a Co-Cr alloy dental prosthesis realized via selective laser melting [2].

	Co	Cr	W	Mo	Si
at%	54.49 ± 1.44	26.15 ± 0.49	9.29 ± 1.54	4.86 ± 0.33	5.21 ± 0.44
wt%	46.65 ± 2.57	19.75 ± 0.92	24.71 ± 3.45	6.76 ± 0.40	2.13 ± 0.20

**Table 2:** Average atomic and mass concentrations with a standard deviation of a Ni-Cr alloy dental prosthesis realized via selective laser melting [2].

	Ni	Cr	Mo	Si	Nb	Al
at%	63.90 ± 1.76	25.74 ± 0.66	6.30 ± 1.15	2.61 ± 0.71	0.82 ± 0.24	0.63 ± 0.44
wt%	64.02 ± 2.11	22.85 ± 0.63	10.30 ± 1.85	1.25 ± 0.33	1.29 ± 0.38	0.29 ± 0.20

**Table 3:** Resulting mechanical characteristics from the indentation tests of the dental prostheses.

Property	Material	Ni-Cr	Co-Cr	Co-Cr	Co-Cr
	Method	SLM	Casting	SLM	SLM
	Post-Process	Finished	Polished	Finished	Polished
Hardness [GPa] $H_{IT}$ 25/40/30/40	1	0.866	1.824	1.607	1.892
	2	0.843	1.648	1.7	2.581
	3	0.815	1.437	1.741	2.252
	Average	0.841	1.636	1.683	2.242
Max. displ. [μm]	1	33.789	18.542	20.921	16.433
	2	35.316	20.177	19.875	12.906
	3	37.13	23.378	19.665	15.278
	Average	35.41	20.699	20.154	14.872
Zero displ. [μm]	1	17.759	6.193	7.147	8.279
	2	17.828	7.077	6.682	4.953
	3	18.138	8.077	6.41	5.883
	Average	17.908	7.116	6.746	6.372
Contact depth [μm]	1	24.369	11.177	12.74	10.763
	2	25.079	12.405	12.02	7.829
	3	26.012	14.289	11.728	9.001
	Average	25.153	12.624	12.163	9.198
Contact stiffness [N/μm]	1	1.903	2.536	2.283	3.293
	2	1.752	2.403	2.378	3.678
	3	1.613	2.053	2.353	2.975
	Average	1.756	2.331	2.338	3.315
Contact area [μm^2^]	1	28,757.482	13,653.115	15,500.097	13,161.51
	2	29,539.568	15,105.065	14,650.44	9646.015
	3	30,562.315	17,315.117	14,305.369	11,056.454
	Average	29,619.788	15,357.766	14,818.635	11,287.993

**Table 4:** Resulting main parameters from zonal nano-roughness determinations on an AFM microscope.

Parameter	Material	Co-Cr	Co-Cr	Co-Cr
	Method	SLM	SLM	Casting
	Post-Process	Finished	Polished	Polished
Average roughness *S_a_* (nm)	1	6.81	4.53	3.37
	2	9.80	2.78	3.72
	3	6.64	2.95	3.50
	Average	7.75	3.42	3.53
Root-mean-square *S_q_* (nm)	1	9.68	5.62	4.30
	2	15.06	3.46	4.81
	3	9.47	3.67	4.50
	Average	11.40	4.25	4.54
Peak-to-peak *S_y_* (nm)	1	94.03	38.09	38.32
	2	183.23	30.94	45.41
	3	94.03	28.56	42.57
	Average	123.76	32.53	42.10

## Data Availability

The data presented in this study are available upon request from the corresponding author.
